# Vaccines Meet Big Data: State-of-the-Art and Future Prospects. From the Classical 3Is (“Isolate–Inactivate–Inject”) Vaccinology 1.0 to Vaccinology 3.0, Vaccinomics, and Beyond: A Historical Overview

**DOI:** 10.3389/fpubh.2018.00062

**Published:** 2018-03-05

**Authors:** Nicola Luigi Bragazzi, Vincenza Gianfredi, Milena Villarini, Roberto Rosselli, Ahmed Nasr, Amr Hussein, Mariano Martini, Masoud Behzadifar

**Affiliations:** ^1^Department of Health Sciences (DISSAL), School of Public Health, University of Genoa, Genoa, Italy; ^2^Department of Experimental Medicine, Unit of Public Health, School of Specialization in Hygiene and Preventive Medicine, University of Perugia, Perugia, Italy; ^3^Unit of Public Health, Department of Pharmaceutical Science, University of Perugia, Perugia, Italy; ^4^Local Health Unit (LHU) ASL3 Genovese, Genoa, Italy; ^5^Department of Medicine and Surgery, Pathology University Milan Bicocca, San Gerardo Hospital, Monza, Italy; ^6^Medical Faculty, University of Parma, Parma, Italy; ^7^Section of History of Medicine and Ethics, Department of Health Sciences, University of Genoa, Genoa, Italy; ^8^Health Management and Economics Research Center, Iran University of Medical Sciences, Tehran, Iran

**Keywords:** vaccine, Big Data, omics disciplines, Web 2.0, eHealth, history of vaccinology

## Abstract

Vaccines are public health interventions aimed at preventing infections-related mortality, morbidity, and disability. While vaccines have been successfully designed for those infectious diseases preventable by preexisting neutralizing specific antibodies, for other communicable diseases, additional immunological mechanisms should be elicited to achieve a full protection. “New vaccines” are particularly urgent in the nowadays society, in which economic growth, globalization, and immigration are leading to the emergence/reemergence of old and new infectious agents at the animal–human interface. Conventional vaccinology (the so-called “vaccinology 1.0”) was officially born in 1796 thanks to the contribution of Edward Jenner. Entering the twenty-first century, vaccinology has shifted from a classical discipline in which serendipity and the Pasteurian principle of the three *I*s (isolate, inactivate, and inject) played a major role to a science, characterized by a rational design and plan (“vaccinology 3.0”). This shift has been possible thanks to Big Data, characterized by different dimensions, such as high volume, velocity, and variety of data. Big Data sources include new cutting-edge, high-throughput technologies, electronic registries, social media, and social networks, among others. The current mini-review aims at exploring the potential roles as well as pitfalls and challenges of Big Data in shaping the future vaccinology, moving toward a tailored and personalized vaccine design and administration.

## Introduction: From the Classical 3Is “Isolate–Inactivate–Inject” Vaccinology 1.0 to Vaccinology 3.0, Vaccinomics, and Beyond

Vaccines are public health interventions aimed at preventing infections-related mortality, morbidity, and disability. As such, they represent a milestone of hygiene and preventive medicine (1). Since their implementation, they have managed to bring several health and economic benefits, both in developed and developing countries, significantly reducing the burden generated by infectious diseases (2). They have contributed to the eradication of smallpox and to the control of others infectious agents, such as polio. According to the estimates of the Global Alliance for Vaccines and Immunization (Gavi), they have contributed to avert up to 23.3 million projected deaths from 2011 to 2020, especially in Africa, Southeast Asia, and in the Eastern Mediterranean (3). Furthermore, they positively impact on perceived quality of life (3) and reduce inequity worldwide (1, 4).

While vaccines have been successfully designed for those infectious diseases preventable by preexisting neutralizing specific antibodies, for other communicable diseases, additional immunological mechanisms should be elicited to achieve a full protection. These additional mechanisms include the stimulation of effector and memory T lymphocytes, besides the release of antibodies by helper T cells-induced B cells (5). A better understanding of immune networks, their sophisticated tuning, and interactions is, as such, fundamental, in those vaccines against HIV/AIDS, malaria or tuberculosis, eluding classical vaccine development, which require new strategies and approaches (6).

“New vaccines” are particularly urgent in the nowadays society, in which economic growth, globalization, and immigration are leading to the emergence/reemergence of old and new infectious agents at the animal–human interface (7, 8).

Conventional vaccinology (the so-called “vaccinology 1.0”) was officially born in 1796 thanks to the contribution of Edward Jenner (1749–1823) and the pioneering discoveries of the New England Puritan minister Cotton Mather (1663–1728), and Lady Mary Wortley Montague (1689–1762), partially anticipated by Chinese and Indians different centuries before. The vaccine typical of vaccinology 1.0 is given by the rabies vaccine, the first human vaccine manufactured in 1885 in the laboratory (9). Other “first generation” vaccines are bacillus Calmette–Guérin (BCG), plague, pertussis, polio, and smallpox vaccines (9).

Entering the twenty-first century, vaccinology has shifted from a discipline in which serendipity and the Pasteurian principle of the three *I*s (isolate, inactivate, and inject) played a major role to a science, characterized by a rational design and plan (10).

If vaccinology 1.0 mainly consisted in isolating infectious agents, cultivating and inactivating them (as a whole or partially), and injecting the obtained product, vaccinology 2.0 utilizes purified microbial cell components. Example of “second generation” vaccines includes vaccines against tetanus, diphtheria, anthrax, pneumonia, influenza, hepatitis B, and Lyme disease (9). The transition from vaccinology 1.0 to vaccinology 2.0 has been made possible by several technological advancements, including genetic and protein engineering, recombinant DNA (11), polysaccharide and carbohydrate chemistry, combinatorial chemistry (12), among others.

Vaccinology 3.0 starts from the microbial genomic sequences (reverse vaccinology 1.0) or from the repertoire of protective human antibodies (reverse vaccinology 2.0) (13, 14). This shift has been possible thanks to omics data, which represent one type of Big Data, characterized by different aspects, such as enormous volume, velocity, and high variety of data (15).

High-throughput technologies-enabled omics disciplines [such as genomics and post-genomics specialties (16, 17), including transcriptomics, proteomics, metabolomics, cytomics, immunomics, secretomics, surfomics, or interactomics], briefly overviewed in Table 1, are able to produce a wealth of data and information, at a large-scale. Recently, these approaches have converged in what is termed vaccinomics, that is, to say the performance of large-scale, hypothesis-free, data-driven and holistic investigations. Poland and collaborators have defined vaccinomics as the “integration of immunogenetics and immunogenomics with systems biology and immune profiling” (18).

**Table 1:** The different genomic/post-genomic specialties and their potential role in the field of vaccinology.

Genomic/post-genomic specialty potentially relevant in vaccinology	Definition
Genomics	Systematic, genome-wide investigation of genes
Proteomics	Systematic, proteome-wide investigation of proteins
Transcriptomics	Systematic, transcriptome-wide investigation of gene transcription
Metabolomics	Systematic, metabolome-wide investigation of metabolites
Cytomics	Systematic, cytome-wide investigation of biochemical/biophysical events at a single cell level
Immunogenomics	Systematic, immunogenome-wide investigation of immunologically relevant genes
Immunoproteomics	Systematic, immunoproteome-wide investigation of immunologically relevant proteins
Immunometabolomics	Systematic, immunometabolome-wide investigation of immunologically relevant metabolites
Interactomics	Systematic, interactome-wide investigation of interactions among proteins and/or other cellular molecules/components
Secretomics	Systematic, secretome-wide investigation of all secreted proteins of a given cell/tissue/organism
Exoproteomics	Systematic, exoproteome-wide investigation of proteins in the extra-cellular proximity of a biological system
Surfomics	Systematic, surfome-wide investigation of surface proteins and other components, such as surface-exposed moieties
Immunomics	Systematic, immunome-wide investigation of immune system dynamics, regulation and response to a given pathogen
Protectomics	Systematic, protectome-wide investigation of the structural/functional protein motifs that confer immunological protection
Adversomics	Systematic, adversome-wide investigation of potential vaccine-related adverse events
Vaccinomics	Systematic, comprehensive integration of previously described omics disciplines for advancing vaccine discovery and development, as well as personalized vaccinology

New cutting-edge technologies include next-generation sequencing (NGS) techniques [RNASeq (19) and large-scale B- and T-cell receptor sequencing (20, 21)], mass cytometry (CyTOF) (22), and peptide/protein arrays (23). Data produced by molecular biology and NGS as well as by bioinformatics (24) can be used to perform mechanistic reductionist studies but can be also exploited to comprehensively capture immune dynamics and interactions (25), carrying out, for instance, network analysis or systems biology (the so-called “systems vaccinology”). Novel bioinformatics tools and new approaches are needed to better integrate the enormous wealth of data originated from omics experiments, making the shift from single-omics to multi-omics possible.

Furthermore, the actual era is characterized by the widespread diffusion of the new information and communication technologies (26): electronic health or eHealth refers to their exploitation as “a means to expand, to assist, or to enhance human activities, rather than as a substitute for them” (27). As omics experiments, eHealth generates as well an enormous wealth of data. Researchers have found that, usually, digital activities correlate with offline behaviors and other variables, such as vaccination knowledge and perception of own risk: for example, Betsch and Wicker (28), investigating a sample of 310 medical students found that explicitly surfing the Internet for vaccination risks-related websites led to fewer public health websites than generically searching for immunization practices.

**Figure 1:**
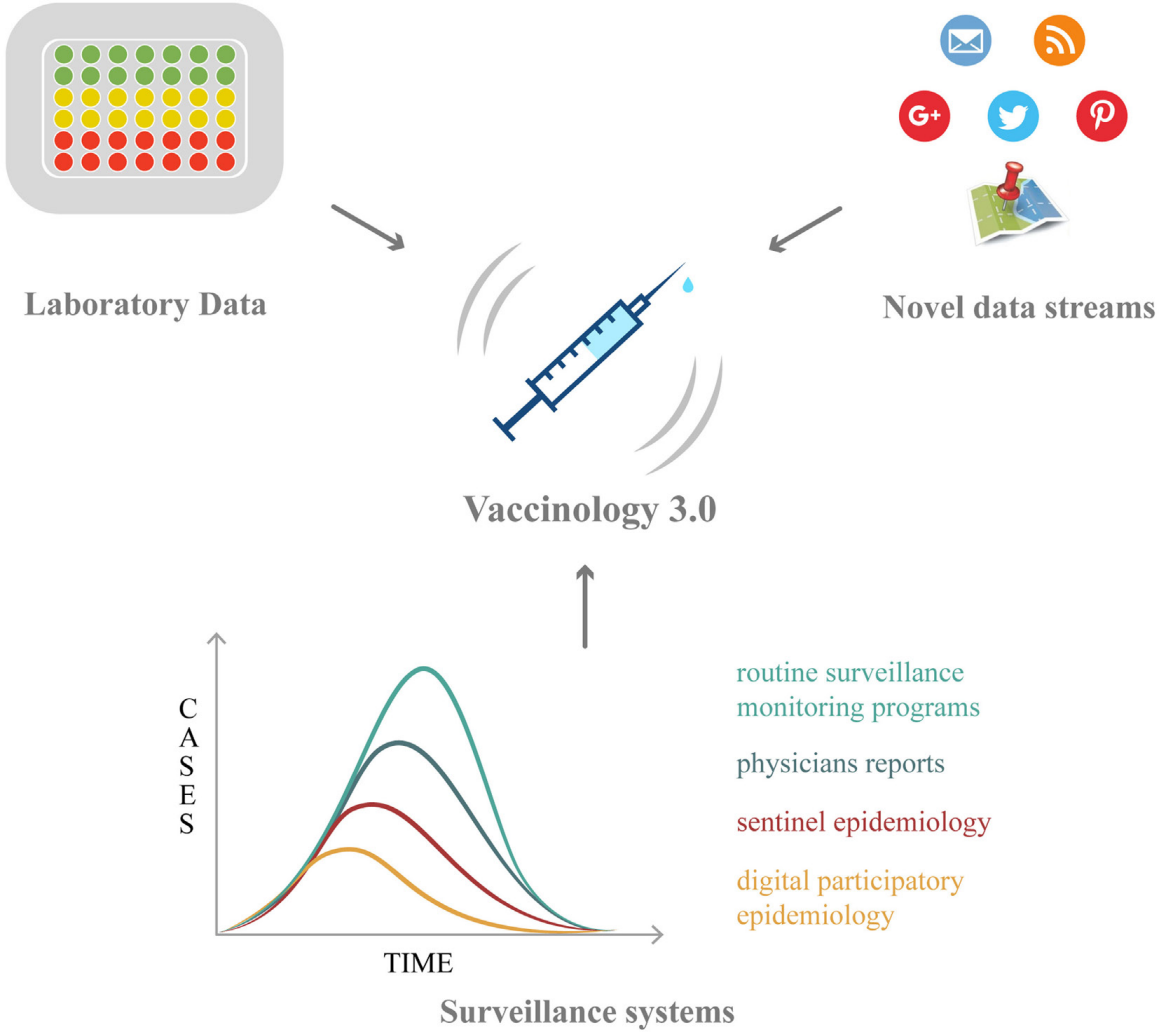
The roles of Big Data in vaccination: from vaccine discovery and development (omics technologies and wet-lab approaches) to vaccination campaign and vaccine safety monitoring (electronic registries, social media/networks, and digital epidemiology).

Vaccinology has now entered a new phase, characterized by new challenges: within this new framework, Big Data hold promises and opportunities, which will be overviewed in the following paragraphs (Table 2; Figure 1).

**Table 2:** Potential applications of Big Data in the different subfields of vaccinology.

Subfield of vaccinology	Examples of applications
Vaccine discovery and development	Structural/functional vaccinologySystems vaccinology Vaccine informatics/bioinformatics*In silico*/computational vaccinology Vaccine ontology Reverse vaccinology Vaccinomics/immunomics

Vaccine production and safety	Monitors and sensors

Vaccine campaigns	Evidence-based prevention and evidence-based vaccinology Immunization registry/information system Personalized vaccinology

Vaccine efficacy and effectiveness	Vaccine trials Vaccine ontology Digital epidemiology/infodemiology and infoveillance

Vaccine side effects	Vaccine adverse event reporting system (VAERS) Vaccine adverse event ontology Adversomics Digital epidemiology/infodemiology and infoveillance

Vaccine literacy/vaccine hesitancy	Digital epidemiology/infodemiology and infoveillance

## Vaccine Discovery and Design: The Role of Big Data

Computational vaccinology (29, 30) and immunoinformatics (31), utilizing algorithms, enable experimental immunology to save time, focusing only on prescreened vaccine candidate antigens and, thus, avoiding cost, time-consuming, and labor intensive steps.

Different *in silico* tools exist, to aid and assist researchers in vaccine discovery and design (32, 33). Databases of vaccine candidates, such as MalVac (34), or Vaxar (35), software tools such as Vaxjo (36), VIOLIN (37–39), NERVE (40), Vaxign (41), Vacceed (42), Jenner-predict server (43), EpiToolKit (44), iVax (45), or VaxiJen (46), have been specifically implemented for vaccinology to enable prediction of vaccine antigens or adjuvants.

A successful example of rationally designed web-based vaccine is the vaccine against *Neisseria meningitidis*, commercially available with the trade name of Bexsero. For the selection of surface antigens, Masignani and collaborators (47) performed genome mining, using computational tools and algorithms, such as PSORT (48), PSI-BLAST (49), and FindPatterns to predict proteins with transmembrane domains, leader peptides, lipo-boxes and outer membrane anchoring motifs. At the end, 570 proteins were selected and GNA1870, a new surface-exposed lipoprotein inducing high levels of bactericidal antibodies, was discovered.

Reverse vaccinology technique is being applied also to other microorganisms, including *Leptospira* (50, 51), *Streptococcus pneumoniae* (52, 53), malaria (54), *Schistosoma* (55), *Echinococcus granulosus* (56), *Rickettsia prowazekii* (57), *Mycobacterium* (58), *Acinetobacter baumannii* (59), *Escherichia coli* (60), *Staphylococcus aureus* (61, 62), *Corynebacterium pseudotuberculosis* (63), *Herpes simplex* (64), *Vibrio cholerae* (65), and *Cryptosporidium* (65, 66), among others.

In the field of veterinary vaccinology, the reverse vaccinology approach is being applied, for instance, to organisms like cattle *neosporosis* (67, 68), *Rhipicephalus microplus* (69–71), *Ehrlichia ruminantium* (72, 73), and bovine herpesvirus 4 (74).

These computational approaches, using massive data mining techniques, rely on brute force (the so-called “test-all-to-lose-nothing” approach). Altindis and collaborators (75) have recently attempted to refine this framework, based on the idea that protective antigens share specific structural/functional features, termed as “protective signatures” or “immunosignatures,” differing from other pathogen components, in terms of immunological properties. Instead of focusing on protein localization, as in previous investigations, Altindis and coworkers concentrated their computational analyses on protein biological role and function. In this sense, their approach, termed as “protectome,” is protein localization unbiased, in that it leads to the identification of surface-exposed and secreted or cytoplasmic protective antigens.

## Big Data and Vaccine Production and Delivery

After production, to properly preserve, store, handle, ship, and deliver vaccine supplies, it is fundamental to maintain cold chain from the manufacturer to the point of use, keeping temperatures within a precise range of values, and avoiding temperature excursions or fluctuations. Vaccines need, indeed, to be stored within a safe zone, namely, between 2 and 8°C (76–78), otherwise their quality is compromised, and their potency cannot be restored. According to the World Health Organization (WHO) and the United Nations Children’s Fund, approximately two percent of health-care facilities in low- and middle-income countries are equipped with proper functional technology for maintaining cold chain.

To solve these issues, Merck and Microsoft have, for example, established a collaboration, in which Merck exploits Microsoft R Server for Hadoop for analyzing, monitoring, and predicting variables that could affect the cold chain, including origin, destination, and delivery route as well as external weather and logistics providers, utilizing special thermal-protection containers equipped with temperature-recording sensors and temperature-sensitive vaccine vial monitors.

Nexleaf has produced ColdTrace (currently, ColdTrace version 5), which has already been implemented in more than 7,000 health-care facilities worldwide, and has recently established a new partnership with www.Google.org and Gavi.

The benefits provided by these technologies are the fact they are low-cost and particularly useful in developing countries, which often rely on stem thermometers or 30-day temperate loggers.

## Big Data and Vaccine Campaigns

Other major sources of Big Data are immunization registries and surveillance systems such as SmiNet-2 (79), or SurvNet@RKI (80). These enormous databases are precious databanks, which can be mined to capture data concerning vaccination coverage rate and its determinants.

Non-conventional data sources or novel data streams, such as Internet search data and tools monitoring web queries, like Google Trends (GT) (81), social media (YouTube, Facebook, Google Plus, Twitter, Pinterest, Instagram, and so on), or news source scraping like HealthMap (82), provide researchers and public health workers with real-time information concerning public reaction to epidemic outbreaks. Novel data streams can track different vaccine-preventable infectious diseases, such as influenza (83–85), pertussis (86, 87), or measles (88), among others. As such, they can be exploited to predict epidemiological figures as well as monitor the effect of vaccine campaigns.

## Big Data and Vaccine Efficacy/Effectiveness

Big Data enable also to individuate molecular signatures and predictors of the outcomes of vaccination, being correlates of vaccine efficacy/effectiveness in different populations (89). Haks and collaborators (89), for instance, utilized transcriptomics to quantitatively assess the immunogenetic signature of immunization response. Dunachie and coworkers (90) explored the differentially expressed genes induced by a malaria candidate vaccine and found that most genes conferring immunological protection belonged to the interferon-gamma and to the proteasome/antigen presentation pathways, differently from genes associated with hemopoietic stem cells, regulatory monocytes, and the myeloid lineage modules.

Novel data streams, such as mobile/smartphone applications, can be utilized in the monitoring and management of vaccine-related data (91).

## Big Data and Vaccine Side Effects

Vaccine adverse events and reactions are very rare. As such, most studies are statistically underpowered to capture the rate of rare/very rare side effects. Meta-analytical approaches and data mining have emerged as useful strategies with this regard. As claimed by Chandler (92), the classical paradigm of the actual pharmacovigilance/vaccine vigilance system based on three stage-approach (namely, signal detection, development of a causality hypothesis, and testing of the causality hypothesis) is plagued by some limitations, in that “routine vaccine pharmacovigilance practice is not sufficient to understand suspected harms that are poorly defined and whose pathophysiology are not completely understood. Furthermore, estimations of risk at the population level fail to acknowledge that vaccines may cause harm in subgroups with individual-level risk factors” for adverse events following immunization. As such new approaches are needed to capture new side effects and, also in this case, Big Data could play a major role.

“Adversomics” is a term coined by Poland in 2009 and is an emerging discipline defined as “the study of vaccine adverse reactions using immunogenomics and systems biology approaches” (93, 94).

Berendsen and coworkers (95) exploited Big Data, to explore BCG-related “non-specific effects,” that is to say effects induced by the vaccination on health beyond its target disease. In particular, they evaluated the effect of timing of BCG on stunting in Sub-Saharan African children under 5 years, analyzing cross-sectional data for 368,450 subjects from 33 controls. Authors found that BCG vaccination did not affect stunting, with timing of BCG vaccination being statistically significant. Similar patterns could be detected for diphtheria–tetanus–pertussis and measles vaccinations.

Vaccine ontology (96, 97), a class of biomedical ontologies, that is to say a consensus-based computer and human interpretable set of terms and relations indicating specific biomedical entities, is another valuable approach. It enables support integrative adverse events-related data collection and analysis, utilizing a normalization strategy more effective than other controlled terminologies. These include the Medical Dictionary for Regulatory Activities, the Common Terminology Criteria for Adverse Events, and the WHO Adverse Reactions Terminology, among others. Using Ontology-Based Vaccine Adverse Event representation, Xie and He (96) explored the adverse events related to Flublok, a recombinant hemagglutinin influenza vaccine.

Novel data streams can be used to see how often people Google for vaccination and for vaccination-related adverse events. Bragazzi and collaborators (98) utilized GT for monitoring the interest toward preventable infections and related vaccines. Authors found that, generally speaking, vaccine was not a popular topic, with the valuable exception of the vaccine against Human Papillomavirus, with vaccines-related queries being approximately one third of the volumes regarding preventable infections. Users tended to search information about possible vaccine-related side effects.

## Big Data and Vaccine Literacy/Vaccine Hesitancy

Big Data enable to track and monitor interest toward vaccination practices (99). The increasing phenomenon of vaccine hesitancy (an umbrella term that includes indecision, uncertainty, delay and reluctance) is multifactorial, and closely linked to social contexts, with different determinants, ranging from geographical area, to political situation, complacency, convenience and confidence in vaccines. Novel data streams, providing a snapshot of perceptions of vaccination in a given place and at a specific time, could be used to assess lay-people’s perceptions of vaccination, enabling health-care workers to actively engage citizens and to plan *ad hoc* communication strategies and plans to contain vaccine hesitancy and to promote vaccine literacy (100).

Shah and colleagues (101) compared time series of rotavirus-related Internet searches as captured by GT with rotavirus laboratory reports from the United States and United Kingdom and with hospitalizations for acute gastroenteritis in the United States and Mexico, before and after national vaccine introductions. Authors found a strong positive correlation between web queries and laboratory reports in the United States (*R*^2^ = 0.79) and United Kingdom (*R*^2^ = 0.60) and between the Internet searches and acute gastroenteritis hospitalizations in the United States (*R*^2^ = 0.87) and Mexico (*R*^2^ = 0.69). Correlations were stronger in the prevaccine period and after vaccine introduction, the mean Internet queries decreased by 40–70% in the United States and Mexico, with a loss of seasonal variation in the United Kingdom.

Bakker and coworkers (102) exploited GT to monitor the interest toward chicken pox, over an 11-year period, from 36 countries. Authors found seasonal peaks with striking latitudinal variation in information seeking behavior. Authors concluded that novel data streams are able to track the global burden of childhood disease as well as to investigate effects of immunization at population level.

Goldlust and collaborators (103) investigated the use of large-scale medical claims data for local surveillance of under-immunization for childhood infections in the United States, developing a statistical framework for integrating disparate data sources on surveillance of vaccination behavior. In this way, authors were able to identify the determinants of vaccine hesitancy behavior. Within the “Vaccine Confidence Project,” Larson and colleagues (104) extensively analyzed data from 10,380 reports (from 144 countries) and found that 7,171 (69%) contained positive or neutral content whereas 3,209 (31%) contained negative content (related to vaccine programs and disease outbreaks, vaccine-related beliefs, awareness, and perceptions; vaccine safety; and vaccine delivery programs).

Within the ambitious “Project Tycho” (freely accessible at www.tycho.pitt.edu) launched by the University of Pittsburgh, United States (105, 106), authors have digitized all weekly surveillance reports of notifiable diseases for United States cities and states published in the period between 1888 and 2011. This data set consists of 87,950,807 reported individual cases and has been used to derive a quantitative history of disease dynamics and transmission in the United States. Pattern analysis has documented, in a statistically robust way, a significant reduction of infections-generated burden, underlining the positive effect of vaccination programs (105). This use of big data emphasizes the dimension of “veracity,” through which is possible to contrast vaccine-related “fake news” and “post-modern, post-factual truths,” disseminated by the anti-vaccination movements (107).

## Conclusion: State-of-the-Art, Current Challenges, and Future Prospects

Big Data have contributed and are expected to continue contributing toward facilitating the discovery, development, production, and delivery of rationally designed vaccines. Further, enabling to identify predictive biomolecular signatures of response to vaccination, vaccination will shift from the classical “one-size-fits-all” paradigm to a personalized approach. Moreover, Big Data can be used to track the success of vaccination campaigns, in term of vaccination coverage rate, as well as the rare/very rate vaccine-related adverse events, for which “classical epidemiological studies” would be statistically underpowered.

However, a number of pitfalls and challenges should be properly recognized to be addressed by future research: Big Data and Big Data sources, as previously overviewed, are highly heterogeneous and should be effectively integrated and harmonized together. Moreover, some algorithms underlying novel data streams need to be refined in that, sometimes, do not exactly predict epidemic outbreaks (108), even though some scholars have shown that, in principle, is possible to correct them to achieve higher predictive power (109). Further, efforts should be done to preserve and protect privacy, confidentiality, and identity. The emerging field of “Big Data Ethics” is trying to address all these issues (110, 111). Currently, we are only witnessing the very beginning of the ongoing “Big Data revolution.”

## Author Contributions

NB, MM, and MB conceived the study. NB, VG, MV, RR, AN, AH, MM, and MB drafted and revised the manuscript; read and approved the last version.

## Conflict of Interest Statement

The authors declare that the research was conducted in the absence of any commercial or financial relationships that could be construed as a potential conflict of interest.

## References

[1] Andre FE Booy R Bock HL Clemens J Datta SK John TJ Vaccination greatly reduces disease, disability, death and inequity worldwide. Bull World Health Organ (2008) 86(2):140–6. 10.2471/BLT.07.040089 18297169 PMC2647387

[2] Lee LA Franzel L Atwell J Datta SD Friberg IK Goldie SJ The estimated mortality impact of vaccinations forecast to be administered during 2011-2020 in 73 countries supported by the GAVI Alliance. Vaccine (2013) 31(Suppl 2):B61–72. 10.1016/j.vaccine.2012.11.035 23598494

[3] Lee BY Bartsch SM Brown ST Cooley P Wheaton WD Zimmerman RK. Quantifying the economic value and quality of life impact of earlier influenza vaccination. Med Care (2015) 53(3):218–29. 10.1097/MLR.0000000000000302 25590676 PMC4690729

[4] Ehreth J The global value of vaccination. Vaccine (2003) 21:596–600. 10.1016/S0264-410X(02)00623-0 12531324

[5] Hagan T Nakaya HI Subramaniam S Pulendran B. Systems vaccinology: enabling rational vaccine design with systems biological approaches. Vaccine (2015) 33:5294–301. 10.1016/j.vaccine.2015.03.072 25858860 PMC4581890

[6] Rappuoli R Aderem A A 2020 vision for vaccines against HIV, tuberculosis and malaria. Nature (2011) 473(7348):463–9. 10.1038/nature10124 21614073

[7] Gutiérrez AH Spero DM Gay C Zimic M De Groot AS. New vaccines needed for pathogens infecting animals and humans: One Health. Hum Vaccin Immunother (2012) 8(7):971–8. 10.4161/hv.20202 22485046

[8] Neiderud CJ. How urbanization affects the epidemiology of emerging infectious diseases. Infect Ecol Epidemiol (2015) 5:27060. 10.3402/iee.v5.27060 26112265 PMC4481042

[9] Rhee JH Towards vaccine 3.0: new era opened in vaccine research and industry. Clin Exp Vaccine Res (2014) 3(1):1–4. 10.7774/cevr.2014.3.1.1 24427757 PMC3890443

[10] Kennedy RB Poland GA The top five “game changers” in vaccinology: toward rational and directed vaccine development. OMICS (2011) 15(9):533–7. 10.1089/omi.2011.0012 21815811 PMC3166183

[11] Lepenies B Yin J Seeberger PH. Applications of synthetic carbohydrates to chemical biology. Curr Opin Chem Biol (2010) 14(3):404–11. 10.1016/j.cbpa.2010.02.016 20227905

[12] Pardee K Slomovic S Nguyen PQ Lee JW Donghia N Burrill D Portable, on-demand biomolecular manufacturing. Cell (2016) 167(1):248–59.e12. 10.1016/j.cell.2016.09.013 27662092

[13] Burton DR. What are the most powerful immunogen design vaccine strategies? Reverse vaccinology 2.0 shows great promise. Cold Spring Harb Perspect Biol (2017) 9(11). 10.1101/cshperspect.a030262 28159875 PMC5540812

[14] Rappuoli R Bottomley MJ D’Oro U Finco O De Gregorio E. Reverse vaccinology 2.0: human immunology instructs vaccine antigen design. J Exp Med (2016) 213(4):469–81. 10.1084/jem.20151960 27022144 PMC4821650

[15] Blohmke CJ O’Connor D Pollard AJ. The use of systems biology and immunological big data to guide vaccine development. Genome Med (2015) 7:114. 10.1186/s13073-015-0236-1 26555212 PMC4641354

[16] Horvatić A Kuleš J Guillemin N Galan A Mrljak V Bhide M. High-throughput proteomics and the fight against pathogens. Mol Biosyst (2016) 12(8):2373–84. 10.1039/c6mb00223d 27227577

[17] Dwivedi P Alam SI Tomar RS. Secretome, surfome and immunome: emerging approaches for the discovery of new vaccine candidates against bacterial infections. World J Microbiol Biotechnol (2016) 32(9):155. 10.1007/s11274-016-2107-3 27465855

[18] Poland GA Ovsyannikova IG Kennedy RB Haralambieva IH Jacobson RM. Vaccinomics and a new paradigm for the development of preventive vaccines against viral infections. OMICS (2011) 15(9):625–36. 10.1089/omi.2011.0032 21732819 PMC3166201

[19] Wang Z Gerstein M Snyder MRNA- Seq: a revolutionary tool for transcriptomics. Nat Rev Genet (2009) 10(1):57–63. 10.1038/nrg2484 19015660 PMC2949280

[20] Yaari G Kleinstein SH. Practical guidelines for B-cell receptor repertoire sequencing analysis. Genome Med (2015) 7:121. 10.1186/s13073-015-0243-2 26589402 PMC4654805

[21] Woodsworth DJ Castellarin M Holt RA. Sequence analysis of T-cell repertoires in health and disease. Genome Med (2013) 5(10):98. 10.1186/gm502 24172704 PMC3979016

[22] Reeves PM Sluder AE Paul SR Scholzen A Kashiwagi S Poznansky MC Application and utility of mass cytometry in vaccine development. FASEB J (2018) 32(1):5–15. 10.1096/fj.201700325R 29092906

[23] Chase BA Johnston SA Legutki JB. Evaluation of biological sample preparation for immunosignature-based diagnostics. Clin Vaccine Immunol (2012) 19(3):352–8. 10.1128/CVI.05667-11 22237890 PMC3294604

[24] He Y Rappuoli R De Groot AS Chen RT. Emerging vaccine informatics. J Biomed Biotechnol (2010) 2010:218590. 10.1155/2010/218590 21772787 PMC3134832

[25] Chattopadhyay PK Roederer M A mine is a terrible thing to waste: high content, single cell technologies for comprehensive immune analysis. Am J Transplant (2015) 15(5):1155–61. 10.1111/ajt.13193 25708158

[26] Bragazzi NL From P0 to P6 medicine, a model of highly participatory, narrative, interactive, and “augmented” medicine: some considerations on Salvatore Iaconesi’s clinical story. Patient Prefer Adherence (2013) 7:353–9. 10.2147/PPA.S38578 23650443 PMC3640773

[27] Oh H Rizo C Enkin M Jadad A. What is eHealth (3): a systematic review of published definitions. J Med Internet Res (2005) 7(1):e1. 10.2196/jmir.7.1.e1 15829471 PMC1550636

[28] Betsch C Wicker S E-health use, vaccination knowledge and perception of own risk: drivers of vaccination uptake in medical students. Vaccine (2012) 30(6):1143–8. 10.1016/j.vaccine.2011.12.021 22192850

[29] Khalili S Jahangiri A Borna H Ahmadi Zanoos K Amani J. Computational vaccinology and epitope vaccine design by immunoinformatics. Acta Microbiol Immunol Hung (2014) 61(3):285–307. 10.1556/AMicr.61.2014.3.4 25261943

[30] Söllner J Heinzel A Summer G Fechete R Stipkovits L Szathmary S Concept and application of a computational vaccinology workflow. Immunome Res (2010) 6(Suppl 2):S7. 10.1186/1745-7580-6-S2-S7 21067549 PMC2981879

[31] Hegde NR Gauthami S Sampath Kumar HM Bayry J The use of databases, data mining and immunoinformatics in vaccinology: where are we? Expert Opin Drug Discov (2018) 13(2):117–30. 10.1080/17460441.2018.1413088 29226722

[32] He Y Xiang Z. Databases and in silico tools for vaccine design. Methods Mol Biol (2013) 993:115–27. 10.1007/978-1-62703-342-8_8 23568467 PMC12060024

[33] He Y Vaccine adjuvant informatics: from data integration and analysis to rational vaccine adjuvant design. Front Immunol (2014) 5:32 10.3389/fimmu.2014.00032 24550916 PMC3909948

[34] Chaudhuri R Ahmed S Ansari FA Singh HV Ramachandran S. MalVac: database of malarial vaccine candidates. Malar J (2008) 7:184. 10.1186/1475-2875-7-184 18811938 PMC2562390

[35] Todd T Dunn N Xiang Z He Y. Vaxar: a web-based database of laboratory animal responses to vaccinations and its application in the meta-analysis of different animal responses to tuberculosis vaccinations. Comp Med (2016) 66(2):119–28. 27053566 PMC4825961

[36] Sayers S Ulysse G Xiang Z He Y. Vaxjo: a web-based vaccine adjuvant database and its application for analysis of vaccine adjuvants and their uses in vaccine development. J Biomed Biotechnol (2012) 2012:831486. 10.1155/2012/831486 22505817 PMC3312338

[37] Xiang Z Todd T Ku KP Kovacic BL Larson CB Chen F VIOLIN: vaccine investigation and online information network. Nucleic Acids Res (2008) 36(Database issue):D923–8. 10.1093/nar/gkm1039 18025042 PMC2238972

[38] He Y Xiang Z. Bioinformatics analysis of *Brucella* vaccines and vaccine targets using VIOLIN. Immunome Res (2010) 6(Suppl 1):S5. 10.1186/1745-7580-6-S1-S5 20875156 PMC2946783

[39] He Y Racz R Sayers S Lin Y Todd T Hur J Updates on the web-based VIOLIN vaccine database and analysis system. Nucleic Acids Res (2014) 42(Database issue):D1124–32. 10.1093/nar/gkt1133 24259431 PMC3964998

[40] Vivona S Bernante F Filippini F NERVE: new enhanced reverse vaccinology environment. BMC Biotechnol (2006) 6:35 10.1186/1472-6750-6-35 16848907 PMC1570458

[41] He Y Xiang Z Mobley HL. Vaxign: the first web-based vaccine design program for reverse vaccinology and applications for vaccine development. J Biomed Biotechnol (2010) 2010:297505. 10.1155/2010/297505 20671958 PMC2910479

[42] Goodswen SJ Kennedy PJ Ellis JT. Vacceed: a high-throughput in silico vaccine candidate discovery pipeline for eukaryotic pathogens based on reverse vaccinology. Bioinformatics (2014) 30(16):2381–3. 10.1093/bioinformatics/btu300 24790156 PMC4207429

[43] Jaiswal V Chanumolu SK Gupta A Chauhan RS Rout C. Jenner-predict server: prediction of protein vaccine candidates (PVCs) in bacteria based on host-pathogen interactions. BMC Bioinformatics (2013) 14:211. 10.1186/1471-2105-14-211 23815072 PMC3701604

[44] Schubert B Brachvogel HP Jürges C Kohlbacher O EpiToolKit – a web-based workbench for vaccine design. Bioinformatics (2015) 31(13):2211–3. 10.1093/bioinformatics/btv116 25712691 PMC4481845

[45] Moise L Gutierrez A Kibria F Martin R Tassone R Liu R iVAX: an integrated toolkit for the selection and optimization of antigens and the design of epitope-driven vaccines. Hum Vaccin Immunother (2015) 11(9):2312–21. 10.1080/21645515.2015.1061159 26155959 PMC4635942

[46] Doytchinova IA Flower DR. VaxiJen: a server for prediction of protective antigens, tumour antigens and subunit vaccines. BMC Bioinformatics (2007) 8:4. 10.1186/1471-2105-8-4 17207271 PMC1780059

[47] Masignani V Comanducci M Giuliani MM Bambini S Adu-Bobie J Arico B Vaccination against *Neisseria meningitidis* using three variants of the lipoprotein GNA1870. J Exp Med (2003) 197(6):789–99. 10.1084/jem.20021911 12642606 PMC2193853

[48] Nakai K Kanehisa M. Expert system for predicting protein localization sites in gram-negative bacteria. Proteins (1991) 11(2):95–110. 10.1002/prot.340110203 1946347

[49] Altschul SF Madden TL Schäffer AA Zhang J Zhang Z Miller W Gapped BLAST and PSI-BLAST: a new generation of protein database search programs. Nucleic Acids Res (1997) 25(17):3389–402. 10.1093/nar/25.17.3389 9254694 PMC146917

[50] Yang HL Zhu YZ Qin JH He P Jiang XC Zhao GP In silico and microarray-based genomic approaches to identifying potential vaccine candidates against *Leptospira* interrogans. BMC Genomics (2006) 7:293. 10.1186/1471-2164-7-293 17109759 PMC1664576

[51] Grassmann AA Kremer FS Dos Santos JC Souza JD Pinto LDS McBride AJA. Discovery of novel leptospirosis vaccine candidates using reverse and structural vaccinology. Front Immunol (2017) 8:463. 10.3389/fimmu.2017.00463 28496441 PMC5406399

[52] Barocchi MA Censini S Rappuoli R. Vaccines in the era of genomics: the pneumococcal challenge. Vaccine (2007) 25(16):2963–73. 10.1016/j.vaccine.2007.01.065 17324490

[53] Talukdar S Zutshi S Prashanth KS Saikia KK Kumar P. Identification of potential vaccine candidates against *Streptococcus pneumoniae* by reverse vaccinology approach. Appl Biochem Biotechnol (2014) 172(6):3026–41. 10.1007/s12010-014-0749-x 24482282 PMC7090528

[54] Tuju J Kamuyu G Murungi LM Osier FHA. Vaccine candidate discovery for the next generation of malaria vaccines. Immunology (2017) 152(2):195–206. 10.1111/imm.12780 28646586 PMC5588761

[55] Merrifield M Hotez PJ Beaumier CM Gillespie P Strych U Hayward T Advancing a vaccine to prevent human schistosomiasis. Vaccine (2016) 34(26):2988–91. 10.1016/j.vaccine.2016.03.079 27036511

[56] Gan W Zhao G Xu H Wu W Du W Huang J Reverse vaccinology approach identify an *Echinococcus granulosus* tegumental membrane protein enolase as vaccine candidate. Parasitol Res (2010) 106(4):873–82. 10.1007/s00436-010-1729-x 20127115

[57] Caro-Gomez E Gazi M Goez Y Valbuena G. Discovery of novel cross-protective *Rickettsia prowazekii* T-cell antigens using a combined reverse vaccinology and in vivo screening approach. Vaccine (2014) 32(39):4968–76. 10.1016/j.vaccine.2014.06.089 25010827 PMC4145598

[58] Pandey K Sharma M Saarav I Singh S Dutta P Bhardwaj A Analysis of the DosR regulon genes to select cytotoxic T lymphocyte epitope specific vaccine candidates using a reverse vaccinology approach. Int J Mycobacteriol (2016) 5(1):34–43. 10.1016/j.ijmyco.2015.10.005 26927988

[59] Hassan A Naz A Obaid A Paracha RZ Naz K Awan FM Pangenome and immuno-proteomics analysis of *Acinetobacter baumannii* strains revealed the core peptide vaccine targets. BMC Genomics (2016) 17(1):732. 10.1186/s12864-016-2951-4 27634541 PMC5025611

[60] Moriel DG Tan L Goh KG Phan MD Ipe DS Lo AW Novel protective vaccine antigen from the core *Escherichia coli* genome. mSphere (2016) 1(6):1–13. 10.1128/mSphere.00326-16 PMC5120174 27904885

[61] Holtfreter S Kolata J Stentzel S Bauerfeind S Schmidt F Sundaramoorthy N Omics approaches for the study of adaptive immunity to *Staphylococcus aureus* and the selection of vaccine candidates. Proteomes (2016) 4(1):1–25. 10.3390/proteomes4010011 28248221 PMC5217363

[62] Oprea M Antohe F. Reverse-vaccinology strategy for designing T-cell epitope candidates for *Staphylococcus aureus* endocarditis vaccine. Biologicals (2013) 41(3):148–53. 10.1016/j.biologicals.2013.03.001 23582120

[63] Soares SC Trost E Ramos RT Carneiro AR Santos AR Pinto AC Genome sequence of *Corynebacterium pseudotuberculosis* biovar *equi* strain 258 and prediction of antigenic targets to improve biotechnological vaccine production. J Biotechnol (2013) 167(2):135–41. 10.1016/j.jbiotec.2012.11.003 23201561

[64] Xiang Z He Y. Genome-wide prediction of vaccine targets for human herpes simplex viruses using Vaxign reverse vaccinology. BMC Bioinformatics (2013) 14(Suppl 4):S2. 10.1186/1471-2105-14-S4-S2 23514126 PMC3599071

[65] Barh D Barve N Gupta K Chandra S Jain N Tiwari S Exoproteome and secretome derived broad spectrum novel drug and vaccine candidates in *Vibrio cholerae* targeted by Piper betel derived compounds. PLoS One (2013) 8(1):e52773. 10.1371/journal.pone.0052773 23382822 PMC3559646

[66] Ifeonu OO Simon R Tennant SM Sheoran AS Daly MC Felix V *Cryptosporidium hominis* gene catalog: a resource for the selection of novel *Cryptosporidium* vaccine candidates. Database (Oxford) (2016) 2016:1–13. 10.1093/database/baw137 28095366 PMC5070614

[67] Goodswen SJ Kennedy PJ Ellis JT. Discovering a vaccine against neosporosis using computers: is it feasible? Trends Parasitol (2014) 30(8):401–11. 10.1016/j.pt.2014.06.004 25028089

[68] Goodswen SJ Kennedy PJ Ellis JT. On the application of reverse vaccinology to parasitic diseases: a perspective on feature selection and ranking of vaccine candidates. Int J Parasitol (2017) 47(12):779–90. 10.1016/j.ijpara.2017.08.004 28893639

[69] Andreotti R Giachetto PF Cunha RC. Advances in tick vaccinology in Brazil: from gene expression to immunoprotection. Front Biosci (Schol Ed) (2018) 10:127–42. 10.2741/s504 28930522

[70] Aguirre Ade A Lobo FP Cunha RC Garcia MV Andreotti R. Design of the ATAQ peptide and its evaluation as an immunogen to develop a *Rhipicephalus* vaccine. Vet Parasitol (2016) 221:30–8. 10.1016/j.vetpar.2016.02.032 27084468

[71] Maritz-Olivier C van Zyl W Stutzer C A systematic, functional genomics, and reverse vaccinology approach to the identification of vaccine candidates in the cattle tick, *Rhipicephalus microplus*. Ticks Tick Borne Dis (2012) 3(3):179–87. 10.1016/j.ttbdis.2012.01.003 22521592

[72] Liebenberg J Pretorius A Faber FE Collins NE Allsopp BA van Kleef M. Identification of *Ehrlichia ruminantium* proteins that activate cellular immune responses using a reverse vaccinology strategy. Vet Immunol Immunopathol (2012) 145(1–2):340–9. 10.1016/j.vetimm.2011.12.003 22261504

[73] Sebatjane SI Pretorius A Liebenberg J Steyn H Van Kleef M. In vitro and in vivo evaluation of five low molecular weight proteins of *Ehrlichia ruminantium* as potential vaccine components. Vet Immunol Immunopathol (2010) 137(3–4):217–25. 10.1016/j.vetimm.2010.05.011 20566221

[74] Palmeira L Machiels B Lété C Vanderplasschen A Gillet L. Sequencing of bovine herpesvirus 4 v.test strain reveals important genome features. Virol J (2011) 8:406. 10.1186/1743-422X-8-406 21846388 PMC3178527

[75] Altindis E Cozzi R Di Palo B Necchi F Mishra RP Fontana MR Protectome analysis: a new selective bioinformatics tool for bacterial vaccine candidate discovery. Mol Cell Proteomics (2015) 14(2):418–29. 10.1074/mcp.M114.039362 25368410 PMC4350036

[76] Lloyd J Cheyne J. The origins of the vaccine cold chain and a glimpse of the future. Vaccine (2017) 35(17):2115–20. 10.1016/j.vaccine.2016.11.097 28364918

[77] Vangroenweghe F. Good vaccination practice: it all starts with a good vaccine storage temperature. Porcine Health Manag (2017) 3:24. 10.1186/s40813-017-0071-4 29214048 PMC5713130

[78] Hatchett R. The medicines refrigerator and the importance of the cold chain in the safe storage of medicines. Nurs Stand (2017) 32(6):53–63. 10.7748/ns.2017.e10960 29094526

[79] Rolfhamre P Jansson A Arneborn M Ekdahl K SmiNet-2: description of an internet-based surveillance system for communicable diseases in Sweden. Euro Surveill (2006) 11(5):103–7. 10.2807/esm.11.05.00626-en 16757847

[80] Faensen D Claus H Benzler J Ammon A Pfoch T Breuer T SurvNet@RKI – a multistate electronic reporting system for communicable diseases. Euro Surveill (2006) 11(4):100–3. 10.2807/esm.11.04.00614-en 16645245

[81] Nuti SV Wayda B Ranasinghe I Wang S Dreyer RP Chen SI The use of Google Trends in health care research: a systematic review. PLoS One (2014) 9(10):e109583. 10.1371/journal.pone.0109583 25337815 PMC4215636

[82] Althouse BM Scarpino SV Meyers LA Ayers JW Bargsten M Baumbach J Enhancing disease surveillance with novel data streams: challenges and opportunities. EPJ Data Sci (2015) 4:17. 10.1140/epjds/s13688-015-0054-0 27990325 PMC5156315

[83] Seo DW Shin SY. Methods using social media and search queries to predict infectious disease outbreaks. Healthc Inform Res (2017) 23(4):343–8. 10.4258/hir.2017.23.4.343 29181246 PMC5688036

[84] Samaras L García-Barriocanal E Sicilia MA. Syndromic surveillance models using web data: the case of influenza in Greece and Italy using Google Trends. JMIR Public Health Surveill (2017) 3(4):e90. 10.2196/publichealth.8015 29158208 PMC5715201

[85] Yang S Santillana M Kou SC. Accurate estimation of influenza epidemics using Google search data via ARGO. Proc Natl Acad Sci U S A (2015) 112(47):14473–8. 10.1073/pnas.1515373112 26553980 PMC4664296

[86] Zhang Y Milinovich G Xu Z Bambrick H Mengersen K Tong S monitoring pertussis infections using Internet search queries. Sci Rep (2017) 7(1):10437. 10.1038/s41598-017-11195-z 28874880 PMC5585203

[87] Pollett S Wood N Boscardin WJ Bengtsson H Schwarcz S Harriman K Validating the use of Google Trends to enhance pertussis surveillance in California. PLoS Curr (2015) 7. 10.1371/currents.outbreaks.7119696b3e7523faa4543faac87c56c2 26543674 PMC4626035

[88] Warren KE Wen LS. Measles, social media and surveillance in Baltimore City. J Public Health (Oxf) (2017) 39(3):e73–8. 10.1093/pubmed/fdw076 27521926

[89] Haks MC Bottazzi B Cecchinato V De Gregorio C Del Giudice G Kaufmann SHE Molecular signatures of immunity and immunogenicity in infection and vaccination. Front Immunol (2017) 8:1563. 10.3389/fimmu.2017.01563 29204145 PMC5699440

[90] Dunachie S Berthoud T Hill AV Fletcher HA. Transcriptional changes induced by candidate malaria vaccines and correlation with protection against malaria in a human challenge model. Vaccine (2015) 33(40):5321–31. 10.1016/j.vaccine.2015.07.087 26256523 PMC4582771

[91] Atkinson KM El-Khatib Z Barnum G Bell C Turcotte MC Murphy MSQ Using mobile apps to communicate vaccination records: a city-wide evaluation with a national immunization app, maternal child registry and public health authorities. Healthc Q (2017) 20(3):41–6. 10.12927/hcq.2017.25289 29132449

[92] Chandler RE Safety concerns with HPV vaccines continue to linger: are current vaccine pharmacovigilance practices sufficient? Drug Safety (2017) 40(12):1167–70. 10.1007/s40264-017-0610-6 28856621 PMC5688196

[93] Poland GA Ovsyannikova IG Jacobson RM Adversomics: the emerging field of vaccine adverse event immunogenetics. Pediatr Infect Dis J (2009) 28(5):431–2. 10.1097/INF.0b013e3181a6a511 19395950 PMC2843136

[94] Whitaker JA Ovsyannikova IG Poland GA. Adversomics: a new paradigm for vaccine safety and design. Expert Rev Vaccines (2015) 14(7):935–47. 10.1586/14760584.2015.1038249 25937189 PMC4630804

[95] Berendsen MLT Smits J Netea MG van der Ven A. Non-specific effects of vaccines and stunting: timing may be essential. EBioMedicine (2016) 8:341–8. 10.1016/j.ebiom.2016.05.010 27428443 PMC4919612

[96] Xie J He Y. Ontology-based vaccine adverse event representation and analysis. Adv Exp Med Biol (2017) 1028:89–103. 10.1007/978-981-10-6041-0_6 29058218

[97] He Y Ong E Xie J Integrative representations and analyses of vaccine-induced intended protective immunity and unintended adverse events using ontology-based and theory-guided approaches. Glob Vaccines Immunol (2016) 1(2):37–9. 10.15761/GVI.1000110 27868103 PMC5111632

[98] Bragazzi NL Barberis I Rosselli R Gianfredi V Nucci D Moretti M How often people Google for vaccination: qualitative and quantitative insights from a systematic search of the web-based activities using Google Trends. Hum Vaccin Immunother (2017) 13(2):464–9. 10.1080/21645515.2017.1264742 27983896 PMC5328221

[99] Amicizia D Domnich A Gasparini R Bragazzi NL Lai PL Panatto D. An overview of current and potential use of information and communication technologies for immunization promotion among adolescents. Hum Vaccin Immunother (2013) 9(12):2634–42. 10.4161/hv.26010 23954845 PMC4162062

[100] Rosselli R Martini M Bragazzi NL. The old and the new: vaccine hesitancy in the era of the Web 2.0. Challenges and opportunities. J Prev Med Hyg (2016) 57(1):E47–50. 27346940 PMC4910443

[101] Shah MP Lopman BA Tate JE Harris J Esparza-Aguilar M Sanchez-Uribe E Use of internet search data to monitor rotavirus vaccine impact in the United States, United Kingdom, and Mexico. J Pediatric Infect Dis Soc (2018) 7(1):56–63. 10.1093/jpids/pix004 28369477 PMC5608630

[102] Bakker KM Martinez-Bakker ME Helm B Stevenson TJ. Digital epidemiology reveals global childhood disease seasonality and the effects of immunization. Proc Natl Acad Sci U S A (2016) 113(24):6689–94. 10.1073/pnas.1523941113 27247405 PMC4914188

[103] Goldlust S Lee E Bansal S Assessing the distribution and drivers of vaccine hesitancy using medical claims data. Online J Public Health Inform (2017) 9(1):e012 10.5210/ojphi.v9i1.7590

[104] Larson HJ Smith DM Paterson P Cumming M Eckersberger E Freifeld CC Measuring vaccine confidence: analysis of data obtained by a media surveillance system used to analyse public concerns about vaccines. Lancet Infect Dis (2013) 13(7):606–13. 10.1016/S1473-3099(13)70108-7 23676442

[105] van Panhuis WG Grefenstette J Jung SY Chok NS Cross A Eng H Contagious diseases in the United States from 1888 to the present. N Engl J Med (2013) 369(22):2152–8. 10.1056/NEJMms1215400 24283231 PMC4175560

[106] Shrestha S Foxman B Berus J van Panhuis WG Steiner C Viboud C The role of influenza in the epidemiology of pneumonia. Sci Rep (2015) 5:15314. 10.1038/srep15314 26486591 PMC4614252

[107] Kata A A postmodern Pandora’s box: anti-vaccination misinformation on the Internet. Vaccine (2010) 28(7):1709–16. 10.1016/j.vaccine.2009.12.022 20045099

[108] Butler D When Google got flu wrong. Nature (2013) 494(7436):155–6. 10.1038/494155a 23407515

[109] Santillana M Zhang DW Althouse BM Ayers JW. What can digital disease detection learn from (an external revision to) Google Flu Trends? Am J Prev Med (2014) 47(3):341–7. 10.1016/j.amepre.2014.05.020 24997572

[110] Milton CL. The ethics of big data and nursing science. Nurs Sci Q (2017) 30(4):300–2. 10.1177/0894318417724474 28934045

[111] Lipworth W Mason PH Kerridge I. Ethics and epistemology of big data. J Bioeth Inq (2017) 14(4):485–8. 10.1007/s11673-017-9815-8 29119459

